# Neutrophil STING is Essential for Heart Repair After Myocardial Infarction/Reperfusion

**DOI:** 10.1016/j.jacbts.2026.101695

**Published:** 2026-10-26

**Authors:** Alma Zernecke, Marie Piollet

**Affiliations:** a Institute of Experimental Biomedicine, University Hospital Würzburg, Würzburg, Germany; b Université Paris Cité, Inserm, PARCC, Paris, France

**Keywords:** inflammation, neutrophils, reperfusion injury

**Figure:**
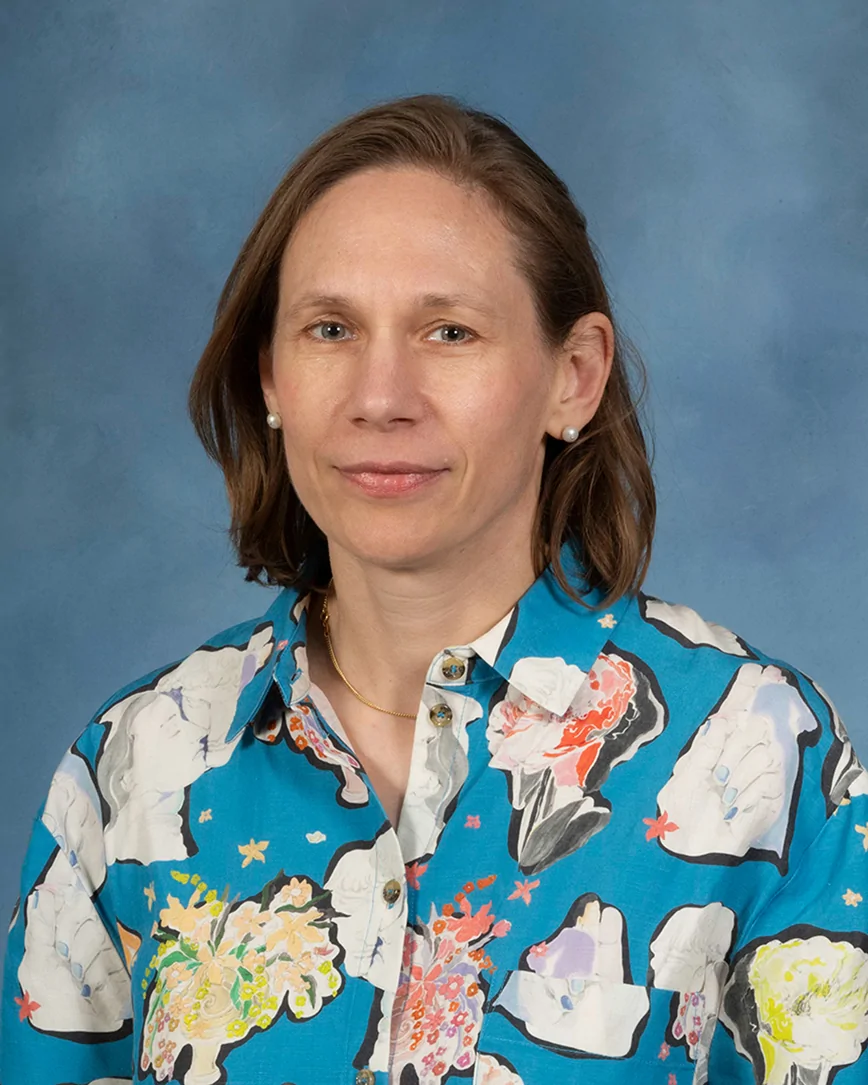

**Figure:**
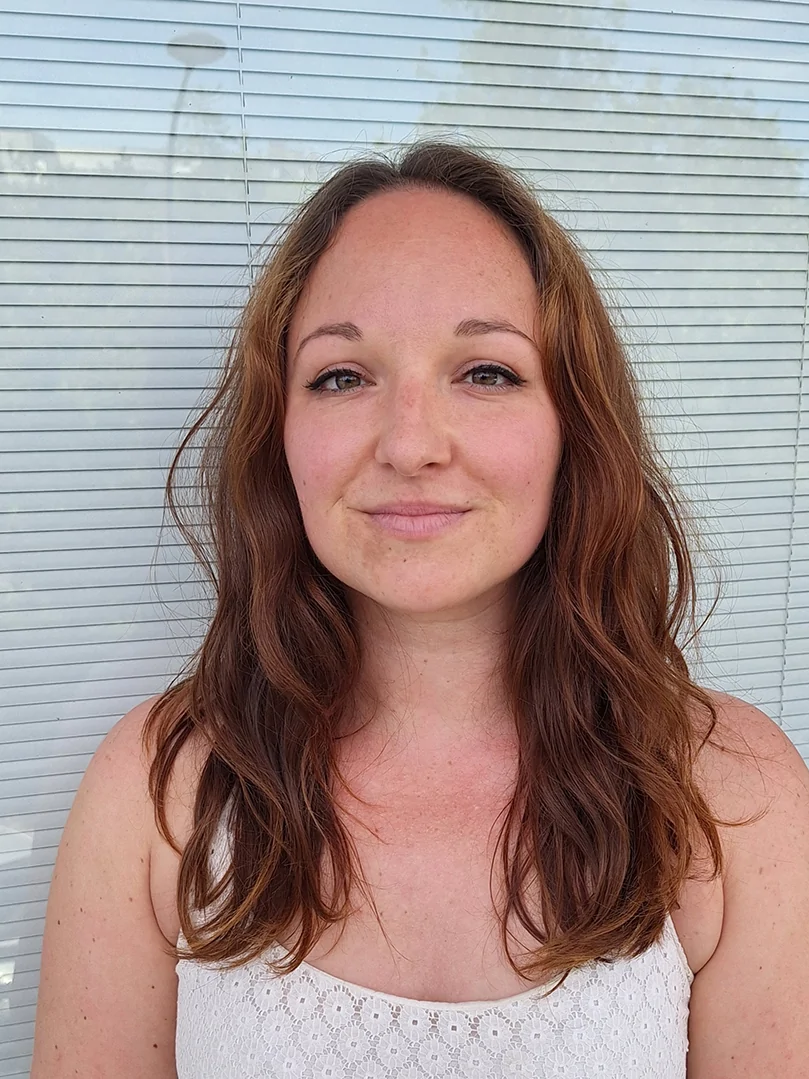

Cardiovascular diseases and myocardial infarction (MI) remain the leading causes of death worldwide. MI triggers a massive inflammatory response in the injured heart. Although critical for effective cardiac healing, inflammation can also cause collateral damage and adverse remodeling, leading to the development of chronic heart failure. The quality of tissue repair is critically influenced by the local immune-inflammatory environment, which is increasingly recognized as a promising therapeutic target to prevent deterioration of heart function. However, despite the promising effects of anti-inflammatory therapies in limiting cardiac injury, they have also yielded contradictory results and major side effects in clinical settings,^1^ highlighting the need for more specific, targeted therapies. Understanding the cellular immune network and its regulation is therefore a prerequisite for developing novel therapeutics.

The type I interferon (IFN) response is a key innate immune response following MI, contributing to inflammatory signaling and tissue remodeling. Type I IFNs and interferon-stimulated genes are induced in multiple cardiac cell populations after MI, including cardiomyocytes in the infarct border zone, fibroblasts, and infiltrating immune cells. Stimulator of Interferon Genes (STING) signaling, activated by the DNA sensor cyclic guanosine monophosphate-adenosine monophosphate synthase (cGAS), has emerged as a key regulator of type I IFN production, as well as mitochondrial function, endoplasmic reticulum stress, and cell survival.^2^^,^^3^

Neutrophils, the first inflammatory cells recruited to the heart after MI, are highly heterogeneous and have multifaceted roles in heart repair.^4^ In this issue of *JACC: Basic to Translational Science*, Brockman et al^5^ identify STING expression in human and murine neutrophils after MI and define its functional role in post-ischemic cardiac repair following MI/reperfusion (R) injury. Unexpectedly, mice with neutrophil-specific STING deficiency (Ly6G^Cre−/+^ STING^flox/flox^) exhibited impaired cardiac function and increased fibrosis, accompanied by elevated type I IFN expression in cardiomyocytes. These findings uncover a previously unrecognized protective role of STING in neutrophils in the response to cardiac injury.

Using single-cell RNA sequencing, the investigators found that STING deficiency broadly reduced neutrophil inflammatory activation and effector functions. STING-deficient neutrophils showed reduced nuclear factor-κB signaling, diminished neutrophil extracellular trap (NET) formation, impaired primary granule release, and decreased phagocytic capacity and reactive oxygen species production. STING deficiency also altered neutrophil trafficking after MI/R. One day after injury, neutrophil-specific STING knockout mice displayed reduced total cardiac neutrophil infiltration. In both the heart and blood, a decrease in C-X-C chemokine receptor 2 neutrophils was observed, and cardiac neutrophils displayed a reduced chemotaxis gene signature, indicating that STING regulates neutrophil recruitment and migratory responses after cardiac injury. In contrast, an expansion of CXCR4+ or CXCR4-expressing neutrophils was observed, which persisted through day 3 post-MI/R, and was attributed to impaired neutrophil turnover. Indeed, STING-deficient neutrophils showed reduced apoptosis in the injured tissue. Macrophages, in turn, engaged in less efferocytosis, which altered macrophage polarization and reduced the number of monocyte-derived anti-inflammatory macrophages. STING signaling in neutrophils thus not only regulated neutrophil function and trafficking but also indirectly shaped the inflammatory environment by influencing macrophage responses.

Collectively, the study by Brockman et al^5^ demonstrates that neutrophil-intrinsic STING signaling is required for effective neutrophil recruitment, inflammatory effector functions, and their timely clearance from the injured myocardium, initiating pro-reparative macrophage polarization. Through these coordinated effects, STING expression in neutrophils is essential for the resolution of inflammation and the transition to cardiac repair, ultimately limiting cardiac dysfunction following MI/R.

The cGAS-STING pathway has largely been considered a pro-inflammatory signaling axis following MI. In cardiomyocytes, activation of STING and downstream type I IFN signaling promotes inflammatory injury and adverse cardiac remodeling. Similarly, suppression of the cGAS-STING pathway promotes the emergence of reparative macrophages,^2^ although recent evidence suggests that macrophage responses are also more nuanced, with monocyte-derived macrophages including a subset of type I IFN-responsive intermediate macrophages that differentiate into major histocompatibility complex class II^hi^ macrophages with cardioprotective functions.^6^ Brockman et al^5^ further extend this concept by demonstrating that the consequences of cGAS-STING activation are highly cell type-specific and exert a protective function in neutrophils. Consistent with this notion, global cGAS or STING deficiency attenuates ischemia-induced emergency granulopoiesis and neutrophil priming in the bone marrow, resulting in reduced neutrophil activation, cytokine production, chemotaxis, phagocytosis, and degranulation, resembling the phenotype reported by Brockman et al.^5^ Despite these effects on neutrophil biology, global cGAS/STING deficiency, however, does not alter cardiac remodeling post-MI.^7^ Together, these findings provide a potential explanation for this seemingly conflicting literature, suggesting that STING signaling has distinct functions, depending on the cellular context. STING signaling in neutrophils is required early after MI to initiate an effective inflammatory response and ensure its timely resolution. In cardiomyocytes, STING activation predominantly promotes inflammatory tissue damage, whereas in macrophages it exerts subset-specific pro- or anti-inflammatory effects.

In neutrophil STING-deficient mice, Brockman et al^5^ observed a marked increase in cardiac fibrosis after MI/R injury. It will be interesting to further understand the mechanistic underpinnings governing modulation of cardiac fibroblasts and myofibroblast phenotypes and whether these effects are directly related to neutrophil functions or to macrophage phenotypic alterations. Indeed, one yet unexplored path would be the potential direct effect of STING-deficient neutrophils on the inflammatory profile of macrophages through paracrine factors, in addition to modulating efferocytosis capacities. For instance, both cell types secrete inflammatory factors such as interleukin-1β and interleukin-6,^8^ which could directly influence fibrosis development.

Recent work has linked mitochondrial dysfunction to type I IFN-mediated cardiac inflammation by showing that loss of the mitochondrial peptide small transmembrane protein 1 disrupts mitochondrial organization, leading to mitochondrial DNA release, cGAS-STING activation, and heart failure during cardiac aging.^9^ Brockman et al^5^ reported up-regulation of mitochondrial genes and oxidative phosphorylation-associated pathways in STING-deficient neutrophils after MI/R, as well as decreased NET production. These observations could suggest a self-amplifying feedback loop in neutrophils, in which mitochondrial damage and NETs activate cGAS-STING signaling, with STING further promoting mitochondrial dysfunction and NET production during cardiac injury.

One major unresolved question raised by the study is why the neutrophil-specific loss of STING has no effect in female mice. Females show a higher type I IFN response,^10^ and this sex-specific difference in interferon responses after MI in both mice and humans complicates translation for clinical purposes. To advance toward modulating the type I IFN response as a therapeutic target for MI, the mechanisms underlying these sex-specific differences need to be elucidated.

Taken together, the study by Brockman et al^5^ highlights the critical role of neutrophils as early pro-inflammatory effector cells that initiate tissue repair following MI/R injury. STING signaling in neutrophils controls neutrophil recruitment and is essential for mounting optimal inflammatory effector functions, processes also required to trigger subsequent clearance and establish a pro-reparative macrophage phenotype. However, these observations should be interpreted within the broader context of cGAS-STING signaling in the heart. Previous studies have reported divergent effects of type I IFN and STING signaling across different cardiac cell types, with global activation promoting inflammation and contributing to pathologic remodeling in conditions such as myocardial infarction, cardiac hypertrophy, and heart failure. Moreover, STING signaling exhibits marked sex-dependent effects. Consequently, therapeutic strategies targeting cGAS-STING will require precise temporal and cell-specific modulation to preserve its beneficial functions while limiting maladaptive inflammatory responses. The cGAS-STING pathway thus constitutes a promising yet incompletely understood therapeutic target, with its context-dependent functions still to be fully elucidated to advance future developments in cardiovascular therapies.

## Funding Support and Author Disclosures

The authors have reported that they have no relationships relevant to the contents of this paper to disclose.

## References

[1] Liberale L., Montecucco F., Schwarz L., Lüscher T.F., Camici G.G. (2021). Inflammation and cardiovascular diseases: lessons from seminal clinical trials. Cardiovasc Res.

[2] Cao D.J., Schiattarella G.G., Villalobos E. (2018). Cytosolic DNA sensing promotes macrophage transformation and governs myocardial ischemic injury. Circulation.

[3] King K.R., Aguirre A.D., Ye Y.X. (2017). IRF3 and type i interferons fuel a fatal response to myocardial infarction. Nat Med.

[4] Piollet M., Grune J., Cochain C. (2026). Neutrophil heterogeneity after myocardial infarction. J Innate Immun.

[5] Brockman M.L., Scruggs T.A., Waller J.P. (2026). STING-expressing neutrophils facilitate resolution of inflammation after myocardial ischemia reperfusion. JACC Basic Transl Sci.

[6] Koenig A.L., Kadyrov F.F., Amrute J.M. (2026). Fate-mapping infiltrating monocytes following experimental myocardial infarction reveals differentiation trajectories in the infarcted heart. J Clin Invest.

[7] Zhu J., Ruan X., Mangione M.C. (2025). The cGAS-STING pathway promotes acute ischemia-induced neutropoiesis and neutrophil priming in the bone marrow. Basic Res Cardiol.

[8] Wynn T.A., Vannella K.M. (2016). Macrophages in tissue repair, regeneration, and fibrosis. Immunity.

[9] Ruberto F.P., Lee C.J.M., Ackers-Johnson M. (2026). Loss of STMP1 perturbs mitochondrial cristae and drives cellular inflammation and heart failure. Circulation.

[10] Gupta S., Nakabo S., Blanco L.P. (2020). Sex differences in neutrophil biology modulate response to type I interferons and immunometabolism. Proc Natl Acad Sci U S A.

